# The Divergence in Bacterial Components Associated with *Bactrocera dorsalis* across Developmental Stages

**DOI:** 10.3389/fmicb.2018.00114

**Published:** 2018-02-01

**Authors:** Xiaofeng Zhao, Xiaoyu Zhang, Zhenshi Chen, Zhen Wang, Yongyue Lu, Daifeng Cheng

**Affiliations:** ^1^Department of Entomology, South China Agricultural University, Guangzhou, China; ^2^Grouped Microorganism Research Center, South China Agricultural University, Guangzhou, China

**Keywords:** *Bactrocera dorsalis*, development stage, microbial community, 16S rRNA, dietary, living environment

## Abstract

Eco-evolutionary dynamics of microbiotas at the macroscale level are largely driven by ecological variables. The diet and living environment of the oriental fruit fly, *Bactrocera dorsalis*, diversify during development, providing a natural system to explore convergence, divergence, and repeatability in patterns of microbiota dynamics as a function of the host diet, phylogeny, and environment. Here, we characterized the microbiotas of 47 *B. dorsalis* individuals from three distinct populations by 16S rRNA amplicon sequencing. A significant deviation was found within the larvae, pupae, and adults of each population. Pupae were characterized by an increased bacterial taxonomic and functional diversity. Principal components analysis showed that the microbiotas of larvae, pupae, and adults clearly separated into three clusters. *Acetobacteraceae, Lactobacillaceae*, and *Enterobacteriaceae* were the predominant families in larval and adult samples, and PICRUSt analysis indicated that phosphoglycerate mutases and transketolases were significantly enriched in larvae, while phosphoglycerate mutases, transketolases, and proteases were significantly enriched in adults, which may support the digestive function of the microbiotas in larvae and adults. The abundances of *Intrasporangiaceae, Dermabacteraceae* (mainly *Brachybacterium*) and *Brevibacteriaceae* (mainly *Brevibacterium*) were significantly higher in pupae, and the antibiotic transport system ATP-binding protein and antibiotic transport system permease protein pathways were significantly enriched there as well, indicating the defensive function of microbiotas in pupae. Overall, differences in the microbiotas of the larvae, pupae, and adults are likely to contribute to differences in nutrient assimilation and living environments.

## Introduction

Many microorganisms reside on the insect exoskeleton, in the gut and hemocoel, and within insect cells (Douglas, 2015), and relationships ranging from parasitism to mutualism are built between microorganisms and insects (Berasategui et al., 2016). These microorganisms are often identified as symbionts of insects (Douglas, 2015). Most of the best-described associations among mutualisms are based on nutritional or defensive services provided by the symbionts to their hosts. The host’s physiology and behavior are often affected in such mutualisms, and the adaptability of the hosts is increased (Ohkuma and Brune, 2011; Ye et al., 2014). The symbionts provide nutrients, such as amino acids and vitamins, or digestive enzymes that aid in the degradation of fastidious dietary polymers or in the detoxification of noxious secondary metabolites (Douglas, 2009). The microorganisms can also protect their host against pathogens, parasites, parasitoids, or predators by producing toxins or antimicrobial compounds for defense (Teixeira et al., 2008; Florez et al., 2015; Hamby and Becher, 2016). Insects can generally employ the symbiont-produced defensive compounds in two different manners: (i) for the protection of the host or its offspring against antagonistic micro- or macroorganisms or (ii) as weed killers in insect fungiculture (Kaltenpoth, 2009; Ramadhar et al., 2014). Antimicrobial compounds produced by the defensive symbiont are of particular importance to insects living in enclosed, humid environments, where opportunistic fungal or bacterial infections can develop rapidly (Douglas, 2015). Studies have recently indicated that metabolic and adaptive abilities allow different bacteria to occupy their host during different host development stages and that the relationships can be multifaceted, varying in their impact on host biology (Lindow and Brandl, 2003; Turnbaugh et al., 2007; Knief et al., 2012). Hosts thus often exploit beneficial symbioses to augment their functional capabilities and to facilitate their adaptation to novel niches (Rio et al., 2006; Ye et al., 2014; Rafael et al., 2016).

For fruit fly, the pivotal roles of microbiota have been identified in recent years, and the factors that affected the structure of microbiota were also investigated. For example, the microbiotas during ontogeny of *Ceratitis capitata* have been reported to be shaped by phylogenetic, metabolic, and taxonomic diversities (Aharon et al., 2013). Some probiotics can even improve the fitness sexual performance of the males at emergence (Hamden et al., 2013). In *Drosophila melanogaster*, symbiotic bacteria play a role in mating preference by changing the levels of cuticular hydrocarbon sex pheromones (Sharon et al., 2010). For *Bactrocera dorsalis*, many studies have identified the structure and function of the gut microbioa. By culture-dependent and the 16S rRNA sequencing methods, the diversity of the cultivable gut bacterial communities associated with *B. dorsalis* have recently been investigated (Wang et al., 2011, 2014; Gujjar et al., 2017), and the development and drug resistantance of *B. dorsalis* were affected by the gut symbionts (Cheng et al., 2017; Khaeso et al., 2017).

Studies have indicated that diet and environment greatly influence the structure of microbiotas (Egert et al., 2004; Antwis et al., 2014; Rebollar et al., 2016). Microbial communities from the surrounding environment can even serve as reservoirs and source pools of colonizers (Kueneman et al., 2014; Loudon et al., 2014). *B. dorsalis* undergoes great changes in living environment during its life, as eggs and larvae in fruit (it is of great possibility to be infected by microbes for *B. dorsalis* larvae in the rotten fruits), pupae (especially in the early stage of pupation) in the ground (enclosed, humid environments, where opportunistic fungal (especially the *Metarhizium* and *Beauveria*) can develop rapidly (Vänninen et al., 2000) and adults on the branches of fruit trees. In addition, larvae must feed on food with high sugar content, the adults must feed on food with high sugar and protein content, and pupae do not eat. And we even found the control efficiency of *Metarhizium* and *Beauveria* to *B. dorsalis* in the pupal stage is very low (data unpublished). These traits may result in differences in the microbiotas of *B. dorsalis*, and *B. dorsalis* may rely on multiple microbial species for fitness and provide a unique model to investigate and compare the population dynamics of symbionts that display varying levels of integration with host biology. Available data on the microbiota of *B. dorsalis* are unfortunately limited, which restricts understanding the microbiota’s influence on host traits, such as diet and living environment.

The larvae and adults of *B. dorsalis* must feed on large amounts of high sugar content food, and the larvae and pupae are exposed to environments with many pathogenic microorganisms. We thus proposed the hypothesis that the symbionts of *B. dorsalis* will change with the development stages: in the larval and adult stages, symbionts promote the host’s absorption of sugar, and some symbionts in the larval and pupal stages may also generate antibiotics to enhance resistance to the pathogens. We explored three questions in the current study. We first examined whether differences in the living environments between adults, larvae, and pupae of *B. dorsalis* are reflected in differences in their bacterial communities, for example more defensive bacteria in larvae and pupae. Second, as larvae and adults must feed on high sugar content food, we tested the hypothesis that functional gene abundances in larval and adult microbiotas would reflect their capacities for sugar and protein metabolism. Finally, we investigated whether functional gene abundances in larval and pupal microbiotas reflect the resistance to pathogens.

## Materials and Methods

### Rearing and Collection of *B. dorsalis*

The lab population of *B. dorsalis* was collected from a carambola (*Averrhoa carambola*) orchard (N 23° 06′ 53.09″, E 113° 24′ 51.29″) in Guangzhou, Guangdong Province in April 2008 and was reared as previously described (Cheng et al., 2017). Briefly, the flies were reared under the following conditions: 25 ± 1°C; 16:8 h light:dark cycle; 70–80% relative humidity (RH). The flies were reared with artificial diets which were treated with high pressure sterilization. Larvae, pupae, and adults of two wild populations were also collected from the cities Huizhou (HZ population) (N 23° 25′ 56.00″, E 114°, 28′ 16.61″) and Nansha (NS population) (N 22° 42′ 25.81″, E 113° 33′ 6.30″) of Guangdong Province in June 2017. For wild populations, carambolas with larvae were collected and take into the lab. Then pupation and eclosion processes went on in the sterile sands. Seventeen samples were collected for the lab population (six larvae, six pupae, and five adults); 15 samples were collected for the HZ and NS population (five larvae, five pupae, and five adults). Each sample consists of one individual.

### Bacterial Community Characterization

All samples (Whole individuals) were selected and then washed with sterile water. The washed samples were transferred to centrifuge tubes containing DNA extraction buffer (with lysozyme) and grinded with a homogenizer. Then total DNA of the samples was extracted using a DNA extraction kit (Tiangen, Beijing, China) following the manufacturer’s instructions. After the DNA of the samples was prepared, qPCR was used to estimate the bacteria absolute content of the samples with the universal bacterial 16S rRNA primers. A standard curve for qPCR was generated by amplifying the 16S rDNA of the *Arthrobacter* sp. isolated from the pupa of *B. dorsalis*. Approximately 465 bp of the V3–V4 region of the bacterial 16S rDNA gene was amplified by PCR according to a standard protocol. The following primers were used: F, 5′-CCTACGGGNGGCWGCAG-3′ and R, 5′-GGACTACHVGGGTATCTAAT-3′. The primers contained the A and B adapters for 454 Life Sciences pyrosequencing and a unique 12-bp error-correcting Golay barcode, which allowed multiplexing of samples in a single run. Each sample was analyzed in a total reaction volume of 25 μL that contained 2.5 μL of Takara 10× Ex Taq buffer, 1.5 μL of Mg^2+^ (25 mM), 2 μL of dNTP (2.5 mM), 0.25 μL of Takara Ex Taq (2.5 U/μL), 0.5 μL of each primer (10 μM), 16.75 μL of ddH_2_O, and 1 μL of template. The PCR amplifications were performed with a 2-min incubation at 95°C followed by 30 cycles of 94°C for 30 s, 57°C for 30 s and 72°C for 30 s, and a final 5-min extension at 72°C. The PCR products were purified using QIAGEN MinElute PCR Purification Kit (QIAGEN, Hilden, Germany) to remove unincorporated primers and nucleotides. A microspectrophotometer ND-1000 (NanoDrop Technologies, Wilmington, DE, United States) was used to measure the concentration of the purified DNA. The purified DNA was sequenced using an Illumina sequencing kit and an Illumina MiSeq sequencer (Illumina, San Diego, CA, United States).

Paired Illumina reads were merged in QIIME (Caporaso et al., 2010). After the high-quality reads were obtained, the data were filtered to remove low-complexity sequences (such as poly-A sequences) and sequences with ambiguous nucleotides, and the operational taxonomic units (OTUs) were picked using USEARCH (Edgar, 2010). The number of OTUs was calculated with mothur software (Schloss et al., 2009) at 97% similarity. An RDP classifier (Huse et al., 2010) was used with naïve Bayes settings for species annotation; the confidence threshold was set to 0.5. Using the species annotations and the read numbers of the OTUs, we generated OTU abundance profiles for all samples. OTUs with an abundance <0.005% of the total data set were removed as an additional level of quality filtering (Bokulich et al., 2013; Navas-Molina et al., 2013).

### Diversity Analyses

Alpha diversity and Shannon rarefaction curves were calculated for all samples in mothur to investigate the species richness of the samples (v.1.34.0) (Schloss et al., 2009). Bray–Curtis and unweighted UniFrac distance matrices were used to calculate the beta diversity and were visualized with principal components analysis (PCA). To determine whether bacterial communities differed among host species, we conducted a shared and unique OTU analysis on the basis of an OTU table generated by QIIME. We used the unweighted pair group method with arithmetic mean (UPGMA), a hierarchical clustering method based on the arithmetic mean, to determine clustering patterns across host species. The UPGMA was used on Bray–Curtis distances of mean OTU relative abundances at the family level. The UPGMA, Bray–Curtis calculations and resulting heatmap were completed using the vegan package (Oksanen et al., 2015) in the R statistical package. Putative microbiota functions were predicted by annotating pathways of OTUs against the KEGG database using PICRUSt (Langille et al., 2013).

### Statistical Analysis

The differences between treatments were compared by a one-way analysis of variance (ANOVA), followed by Tukey’s test for multiple comparisons. The differences were considered significant at the *P* < 0.05 level. The data were analyzed using SPSS software. Analysis of similarity (ANOSIM) for the bacterial community of *B. dorsalis* across developmental stages were done with PRIMER 7.0.

## Results

### Symbionts Content Quantification and Sequencing Data of 16S rRNA

Absolute content of the symbionts in flies were identified with qPCR, the results showed the symbionts content in each individual was about 10^6^ CFU and there is no difference for the absolute content of the symbionts in different development stage (lab: *F*_2,14_ = 0.126, *P* = 0.883; Huizhou: *F*_2,12_ = 0.717, *P* = 0.508; and Nansha: *F*_2,12_ = 1.768, *P* = 0.212) (**Supplementary Figure S1**). After the sequencing data were subjected to demultiplexing, quality filtering and chimera removal, 50082–57433 reads were retained for the 17 lab population samples, 50176–57172 reads were obtained for the 15 YC samples, and 50037–56599 reads were obtained for the 15 NS samples (**Supplementary Table S1**). Shannon rarefaction curves for all samples showed a plateau stage, indicating adequate sampling of 16S rRNA sequences for all the samples (**Supplementary Figure S2**).

### Differences in Larval, Pupal, and Adult Bacterial Communities

Significantly more OTUs were generated in the pupal samples of the three populations (lab: *F*_2,14_ = 30.387, *P* < 0.001; Huizhou: *F*_2,12_ = 5.746, *P* = 0.018; and Nansha: *F*_2,12_ = 5.116, *P* = 0.025) (**Supplementary Figure S3**). The ACE value and the Shannon and Simpson indices indicated that pupae exhibited the greatest species richness and that the richness of adults and larvae did not significantly differ (**Supplementary Table S2**). A major trend is clearly seen during development stages: the bacterial diversities of all populations were closest, on average, during the feeding stage (larvae and adults), while pupae in complex environments showed greater bacterial diversity (*P* < 0.01).

**FIGURE 1:**
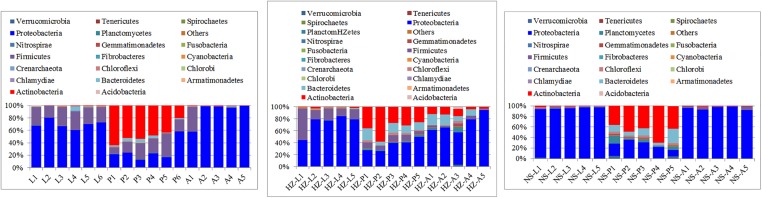
Taxonomic compositions of microbiotas at the phylum level. Bars show proportions of taxa per individual as averaged across conspecifics and estimated from the rarefied OTU table. ‘Others’ group shows all phyla with relative abundance below 1% over the total number of reads. Lab population (L: larvae, P: pupae, A: adults); Huizhou population (HZ-L: larvae, HZ-P: pupae, HZ-A: adults); and Nansha population (NS-L: larvae, NS-P: pupae, NS-A: adults).

Larvae and adults were significantly enriched in *Proteobacteria* at the phylum level more than pupae (for larvae: a cumulative relative abundance above 60, 44.3, and 91.9% in larvae for the lab, Huizhou and Nansha populations, respectively, *P* < 0.01; and for adults: a cumulative relative abundance above 57.82, 54.32, and 92.12% for the lab, Huizhou and Nansha populations, respectively, *P* < 0.01). *Actinobacteria* had a cumulative relative abundance in pupae above 20.54, 26.01, and 19.23% for the lab, Huizhou and Nansha populations, respectively (**Figure 1**).

**FIGURE 2:**
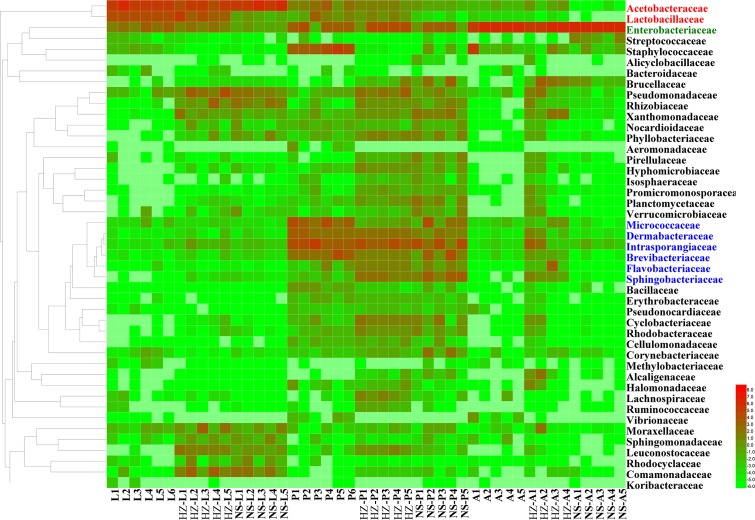
Unweighted pair group method with arithmetic mean (UPGMA) and heatmap of bacterial families with relative abundances across different samples. Rows are bacterial families. Columns are samples. Colors indicate taxa with a higher (red) or lower (green) relative abundance in each sample. Taxonomic units in red, green and blue have significantly higher abundances in larvae, adults and pupae, respectively. Lab population (L: larvae, P: pupae, A: adults); Huizhou population (HZ-L: larvae, HZ-P: pupae, HZ-A: adults); and Nansha population (NS-L: larvae, NS-P: pupae, NS-A: adults).

The abundance of the dominant OTUs in each stage was compared with that in the other two stages (**Figure 2** and **Supplementary Data Sheet S1**).

For lab population, the most abundant OTUs (average relative abundance ± SD between replicates) in larvae were *Acetobacteraceae* (*Acetobacter* sp.) (54.62 ± 8.47), *Lactobacillaceae* (*Lactobacillus brevis*) (24.49 ± 3.88), *Enterobacteriaceae* (4.94 ± 2.2) and *Acetobacteraceae* (*Gluconobacter* sp.) (4.77 ± 0.88); in pupae, the most abundant OTUs were *Micrococcaceae* (12.15 ± 8.54), *Intrasporangiaceae* (7.03 ± 4.97), *Brevibacteriaceae* (mainly *Brevibacterium*) (6.51 ± 6.28), and *Dermabacteraceae* (mainly *Brachybacterium*) (5.36 ± 3.01); in adults the most abundant OTU was *Enterobacteriaceae* (84.74 ± 18.11) (**Figure 2** and **Supplementary Data Sheet S1**).

For Huizhou population, the most abundant OTUs in larvae were *Acetobacteraceae* (*Acetobacter* sp.) (20.25 ± 3.11) and *Lactobacillaceae* (*Lactobacillus brevis*) (10.08 ± 3.48); the most abundant OTUs in pupae belonged to *Intrasporangiaceae* (10.79 ± 1.53), *Dermabacteraceae* (mainly *Brachybacterium*) (4.03 ± 2.22), and *Brevibacteriaceae* (mainly *Brevibacterium*) (1.63 ± 0.97); and the most abundant OTU in adults was *Enterobacteriaceae* (26.96 ± 9.52) (**Figure 2** and **Supplementary Data Sheet S1**).

For Nansha population, the most abundant OTUs in larvae were *Acetobacteraceae* (*Acetobacter* sp.) (54.04 ± 10.66); in pupae, the most abundant OTUs were *Intrasporangiaceae* (14.72 ± 1.65), *Dermabacteraceae* (mainly *Brachybacterium*) (2.36 ± 0.83), and *Brevibacteriaceae* (mainly *Brevibacterium*) (1.23 ± 0.67); and the most abundant OTU in adults was *Enterobacteriaceae* (18.89 ± 7.08) (**Figure 2** and **Supplementary Data Sheet S1**).

In conclusion, we found *Acetobacteraceae* (*Acetobacter* sp.) was the most abundant OTU in larvae of the three populations; *Intrasporangiaceae, Dermabacteraceae* (mainly *Brachybacterium*), and *Brevibacteriaceae* (mainly *Brevibacterium*) were the most abundant OTUs in pupa of the three populations; *Enterobacteriaceae* was the most abundant OTU in adult of the three populations (**Figure 2** and **Supplementary Data Sheet S1**).

**FIGURE 3:**
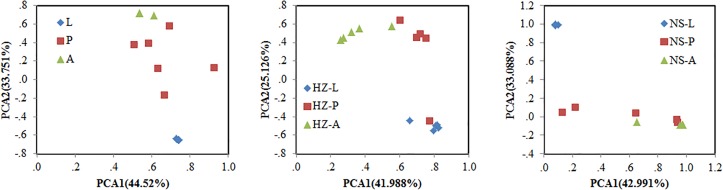
Principal components analysis (PCA) of bacterial communities (genus level) according to development stages of the three populations (lab, Huizhou and Nansha populations). Taxonomic (OTU) clustering based on unweighted UniFrac distances. Lab population (L: larvae, P: pupae, A: adults); Huizhou population (HZ-L: larvae, HZ-P: pupae, HZ-A: adults); and Nansha population (NS-L: larvae, NS-P: pupae, NS-A: adults).

**FIGURE 4:**
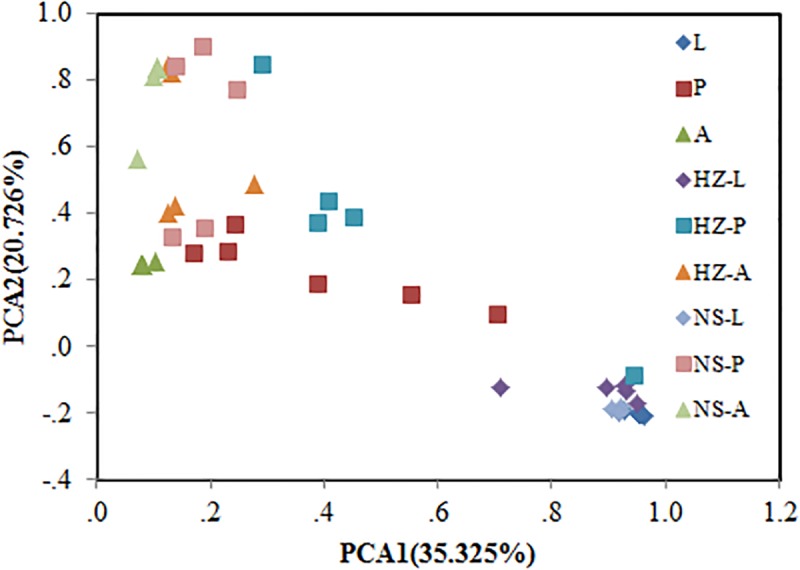
Bray–Curtis distances-based PCA of all samples of the three populations (lab, Huizhou and Nansha populations). Lab population (L: larvae, P: pupae, A: adults); Huizhou population (HZ-L: larvae, HZ-P: pupae, HZ-A: adults); and Nansha population (NS-L: larvae, NS-P: pupae, NS-A: adults).

Bacterial communities of larvae, pupae, and adults showed a clear pattern of specialization based on unweighted UniFrac distances with OTUs annotated at the genus level (PCA, **Figure 3**), indicating the specialization of larvae, pupae, and adults in hosting OTUs unique to each stage. PCA was also used to compare the similarity in the microbial community compositions of all samples of the three populations. Larvae, pupae, and adults each formed a distinct cluster among all samples, and these three clusters were separated from each other (**Figure 4**). The clustering pattern among samples was not influenced by the sampling population, as samples from the three populations clustered together, with the exception that results from pupal samples formed two different clusters. Moreover, ANOSIM results indicated that there were significant differences in the bacterial community of *B. dorsalis* across developmental stages (lab: *R* = 0.998, *P* = 0.001; Huizhou: *R* = 0.745, *P* = 0.001; and Nansha: *R* = 0.951, *P* = 0.001).

### Functional Prediction of Larval, Pupal, and Adult Microbiotas

**FIGURE 5:**
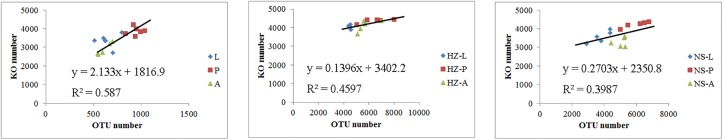
Pearson relationship analysis of KO number and OTU number. Lab population (L: larvae, P: pupae, A: adults); Huizhou population (HZ-L: larvae, HZ-P: pupae, HZ-A: adults); and Nansha population (NS-L: larvae, NS-P: pupae, NS-A: adults).

**FIGURE 6:**
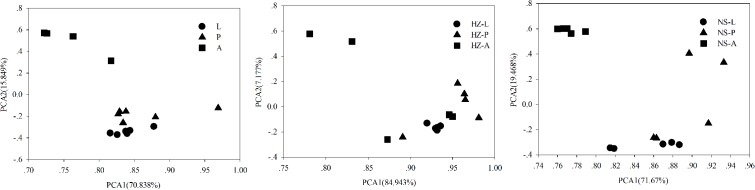
Principal components analysis of functional diversity according to development stages of the three populations (lab, Huizhou and Nansha populations). Lab population (L: larvae, P: pupae, A: adults); Huizhou population (HZ-L: larvae, HZ-P: pupae, HZ-A: adults); and Nansha population (NS-L: larvae, NS-P: pupae, NS-A: adults).

We addressed whether increased OTU diversity confers the host with a higher functional diversity. We predicted 4364, 4590, and 4308 level 3 KEGG Orthology (KO) groups in the predicted metagenomes of the lab, Huizhou and Nansha populations, respectively (PICRUSt analysis, **Supplementary Data Sheet S2**). The pattern of functional diversity largely followed the trend in taxonomic diversity: pupae displayed greater bacterial taxonomic and functional diversity than larvae and adults (lab: *R*^2^ = 0.587, *P* < 0.01; Huizhou: *R*^2^ = 0.678, *P* = 0.005; and Nansha: *R*^2^ = 0.3987, *P* = 0.012; Pearson relationship, **Figure 5**). The microbial community clusters of larvae, pupae, and adults were significantly separated at both the OTU and KO levels (**Figures 3, 6**), indicating a strong development effect.

**FIGURE 7:**
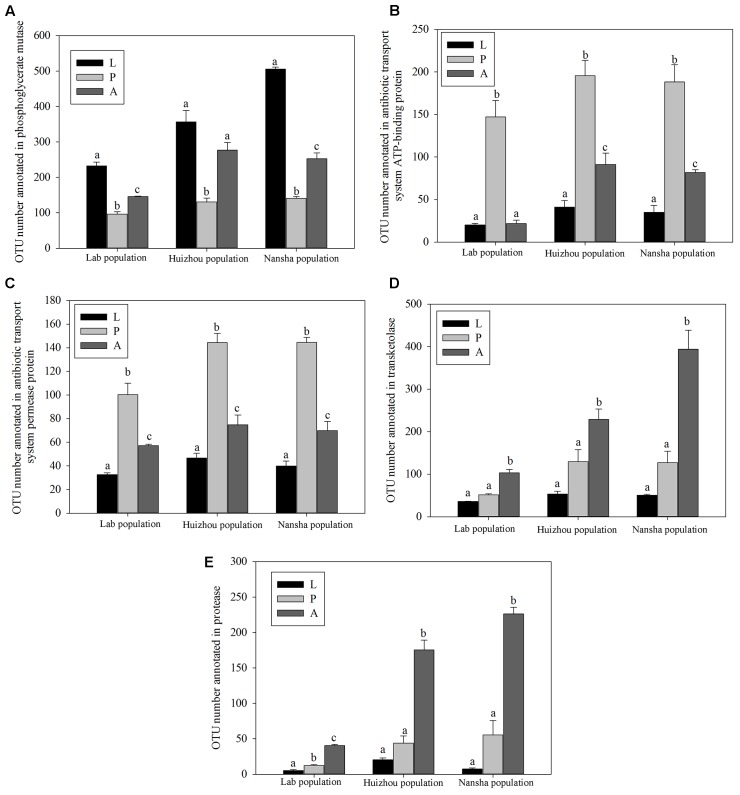
Comparison of predicted KEGG ortholog counts. Means ± SEMs labeled with different letters are significantly different. OTU numbers annotated for phosphoglycerate mutase **(A)**, antibiotic transport system ATP-binding proteins **(B)**, antibiotic transport system permease proteins **(C)**, transketolase **(D)**, and protease **(E)**.

PICRUSt analysis predicted that phosphoglycerate mutase was significantly more abundant in larvae and adults than in pupae (lab population: *F*_2,14_ = 81.873, *P* < 0.01; Huizhou: *F*_2,12_ = 25.055, *P* < 0.01; and Nansha: *F*_2,12_ = 307.792, *P* < 0.01; **Figure 7A**). Phosphoglycerate mutase is a key enzyme in glucose metabolism and involved in converting 3-phosphoglyceric acid into 2-phosphoglyceric acid. Significantly greater number of OTUs were annotated for “antibiotic transport system ATP-binding proteins” and “antibiotic transport system permease proteins” in pupae (for antibiotic transport system ATP-binding proteins, lab population: *F*_2,14_ = 37.387, *P* < 0.01; Huizhou: *F*_2,12_ = 32.737, *P* < 0.01; and Nansha: *F*_2,12_ = 37.762, *P* < 0.01; for antibiotic transport system permease proteins, lab population: *F*_2,14_ = 33.668, *P* < 0.01; Huizhou: *F*_2,12_ = 52.155, *P* < 0.01; and Nansha: *F*_2,12_ = 89.963, *P* < 0.01; **Figures 7B,C**). And significantly greater number of OTUs were annotated for transketolase, which is involved in the pentose phosphate pathway, in adults (lab population: *F*_2,14_ = 67.256, *P* < 0.01; Huizhou: *F*_2,12_ = 16.496, *P* < 0.01; and Nansha: *F*_2,12_ = 35.811, *P* < 0.01; **Figure 7D**). Moreover, a significantly greater number of OTUs were annotated with the protease involved in the protein metabolism pathway in adults, which may indicate a function in protein metabolism (lab population: *F*_2,14_ = 140.849, *P* < 0.01; Huizhou: *F*_2,12_ = 69.395, *P* < 0.01; and Nansha: *F*_2,12_ = 78.369, *P* < 0.01; **Figure 7E**).

## Discussion

Although studies on fly microbiotas have been reported (Aharon et al., 2013; Aksoy et al., 2014; Augustinos et al., 2015; Michael et al., 2016; Cheng et al., 2017; Yong et al., 2017b; Zhao et al., 2017), little is known about the microbial community differences during different development stages, and the former studies mainly focused on the gut microbiotas of the flies (Aksoy et al., 2014; Augustinos et al., 2015; Michael et al., 2016; Cheng et al., 2017; Zhao et al., 2017). The number of OTUs observed in the larvae, pupae, and adults of *B. dorsalis* in this study is greater than those reported in other fly gut samples (**Figure 1**), suggesting that the function of the microbial community in *B. dorsalis* must be analyzed in detail. Actually microbiomes associated with *B. dorsalis* have been reported in earlier studies. For example, Andongma et al. (2015) have reported that the dominance of *Firmicutes* in adult stages and *Proteobacteria* in immature stages (Andongma et al., 2015); Wang et al. (2011) and Yong et al. (2017b) revealed Proteobacteria (specifically Gammaproteobacteria) to be predominant in the male adults of *B. dorsalis* (Wang et al., 2011; Yong et al., 2017b). In our study *Proteobacteria* was the dominant phylum in larvae and adults, which is consistent with previous studies (Aksoy et al., 2014; Yong et al., 2017b); however, *Actinobacteria* were mainly found in pupae, which may indicate that the pupal microbiota has a different function. Although Andongma et al. (2015) investigated symbiotic bacterial populations across life stages of *B. dorsalis*, many fewer OTUs were identified in their study; moreover, fewer *Actinobacteria* were not identified in pupa in their study (Andongma et al., 2015). The DNA extraction method may be the key reason for differences between their study and the current study. DNA is difficult to extract from *Actinobacteria* without lysozyme digestion because of its special cell wall (Zhang et al., 2013). Andongma et al. (2015) did not digest the sample with lysozyme, possibly causing the absence of *Actinobacteria* contributions. Moreover, they used pupae without puparium, which may explain the lack of *Actinobacteria* since bacteria of this order are often located in specific regions of the surface of insect hosts (Kaltenpoth, 2009). The greater sequencing depth in our study might also explain the greater number of identified OTUs since we obtained many more reads than Andongma reported. Actually other study has also identified *Actinobacteria* (Yong et al., 2017b).

Insects show remarkable adaptations to exploit diverse nutritional resources; these adaptations are due to the wide diversity of digestive enzymes produced by the insects themselves as well as the metabolic capabilities of symbiotic microorganisms that overcome the host’s nutritional limitations (Berasategui et al., 2016). The high abundance of *Proteobacteria* observed in larvae and adults likely supports their importance in sugar metabolism. *Acetobacteraceae, Lactobacillaceae*, and *Enterobacteriaceae* were the most dominant families within this phylum that were observed in all larval and adult samples and have been reported to function in sugar metabolism (Kersters et al., 2006; Lambert et al., 2011; Yong et al., 2017a). However, we found that *Acetobacteraceae* and *Lactobacillaceae* were the most dominant families in larvae, while *Enterobacteriaceae* was the most dominant family in adults. This result may suggest that the digestion process may differ in larvae and adults, resulting in changes in the microbiota composition. Unlike the larvae, the adults must also feed on a high-protein diet, and protein in their diet can even influence the mating behavior of flies (Shelly and Kennelly, 2002; Shelly et al., 2005). An important family that is associated with most fruit flies is *Enterobacteriaceae*; members of this group play very important roles in courtship and reproduction (Ben Ami et al., 2010). We also found that pathways involved in protein metabolism were significantly enriched in microbiotas in adults. These results indicate that microbiotas may also be involved in digesting protein in food. More evidence is needed to prove whether members of *Enterobacteriaceae* also play a role in protein digestion to influence the courtship and reproduction of *B. dorsalis*. Newell et al. (2014) also reported that some *Acetobacteraceae* species in the gut of *Drosophila* were involved in oxidative stress detoxification and encoded an efflux pump (Newell et al., 2014). Researches have suspected that *Lactobacillaceae* and *Enterobacteriaceae* contribute to digestion and protection against parasites and pathogens in insect gut (Billiet et al., 2017; Smith et al., unpublished). We thus need detailed investigation of the specific bacteria of *B. dorsalis* by pure culture methods.

Insect-associated microbes are just beginning to be exploited as promising sources of novel bioactive compounds (Dettner, 2011). Microbial symbionts providing chemical defense for the host against predators, parasites, parasitoids, and pathogens occur in several insect taxa, including beetles (Kellner, 2002), psyllids (Nakabachi et al., 2013), planthoppers (Fredenhagen et al., 1987), and solitary wasps (Kaltenpoth et al., 2005; Kaltenpoth, 2009; Kaltenpoth and Engl, 2014). The high abundance of *Actinobacteria* observed in the pupae of *B. dorsalis* support their importance in producing compounds with antimicrobial activity. *Actinobacteria* are known to be important sources suited as defensive symbionts of insects (Kaltenpoth, 2009). The number of OTUs in pupae that represent antibiotic transport system ATP-binding and antibiotic transport system permease proteins is significantly greater than that in larvae and adults, which strongly supports the defensive function of *Actinobacteria* in the pupae of *B. dorsalis*. *Actinobacteria* are particularly common and widespread in soil (Goodfellow and Williams, 1983) and are therefore regularly encountered by insects living in soil. The pupae of *B. dorsalis* consistently remained in soil until emergence. Kaltenpoth (2009) stated that three key factors probably contribute to the role of *Actinobacteria* as defensive exosymbionts in insects: (i) their ability to utilize a wide variety of carbon sources and to generally subsist on low levels of resources; (ii) the capacity of some taxa to form spores and thereby survive inhospitable conditions in the soil; and (iii) their ability to produce secondary metabolites with antibiotic properties (Goodfellow and Williams, 1983). The evolution of symbiotic interactions between *Actinobacteria* and insects might have been initiated by commensal or facultative entomopathogenic *Actinobacteria* that exploited the low amounts of compounds present on the cuticle or in the excretions of soil-dwelling insects. Once the bacteria became associated with an insect, antibiotic substances produced for the microbes’ own protection might have also become beneficial for the host insect (Kaltenpoth, 2009). The specific inhabitation of *Intrasporangiaceae, Dermabacteraceae*, and *Brevibacteriaceae* in pupae in our study may indicate their defensive function. The defensive function of bacteria in *Brevibacteriaceae* has been previously revealed by pure culture methods, and researchers have identified the relevant bacteria and antibacterial compound (Ryser et al., 1994; Maisnierpatin and Richard, 1995). *Brachybacterium* of *Dermabacteraceae* were also identified to express strong antimicrobial activity (Liu et al., 2011). Pupae can therefore be used in future studies as a source from which *Actinomycetes* with antimicrobial activity can be isolated.

## Conclusion

The larvae, pupae, and adults of *B. dorsalis* were observed to harbor distinct microbial flora. We performed a detailed investigation of the microbial flora of *B. dorsalis* that provides a basis for future research. Further studies to investigate the microbial composition may provide a comprehensive understanding of the differences in diet and physiological behavior among *B. dorsalis*. Moreover, host-specific microbial species, for example, those from the phylum *Actinobacteria*, can be used to develop potential compounds with antimicrobial activity that have potential value for human application.

## Availability of Data and Material

Sequence data has been deposited at NCBI under Bioproject PRJNA415228.

## Author Contributions

Conceived and designed the experiments: DC; analyzed the data: DC and YL; contributed reagents/materials/analysis tools: XfZ, XyZ, ZC, and ZW; wrote the paper: DC.

## Conflict of Interest Statement

The authors declare that the research was conducted in the absence of any commercial or financial relationships that could be construed as a potential conflict of interest.

## Supplementary Material

FIGURE S1 Absolute content of symbionts in flies.

FIGURE S2 Shannon rarefaction curves for all samples.

FIGURE S3 Differences between the OTU numbers of the samples.

TABLE S1 Summary of 16S rDNA read counts for all samples of *B. dorsalis*.

TABLE S2 Comparison of OTU diversity of 16S rRNA gene amplicons.

DATA SHEET S1 Abundance of the dominant OTUs.

DATA SHEET S2 KEGG Orthology groups predicted for the samples.

## References

[B1] Aharon Y. Pasternak Z. Ben Yosef M. Behar A. Lauzon C. Yuval B. (2013). Phylogenetic, metabolic, and taxonomic diversities shape mediterranean fruit fly microbiotas during ontogeny. *Appl. Environ. Microbiol.* 79 303–313. 10.1128/AEM.02761-12 23104413 PMC3536086

[B2] Aksoy E. Telleria E. L. Echodu R. Wu Y. Okedi L. M. Weiss B. L. (2014). Analysis of multiple tsetse fly populations in Uganda reveals limited diversity and species-specific gut microbiota. *Appl. Environ. Microbiol.* 80 4301–4312. 10.1128/AEM.00079-14 24814785 PMC4068677

[B3] Andongma A. A. Wan L. Dong Y. C. Li P. Desneux N. White J. A. (2015). Pyrosequencing reveals a shift in symbiotic bacteria populations across life stages of *Bactrocera dorsalis*. *Sci. Rep.* 5:9470. 10.1038/srep09470 25822599 PMC5380164

[B4] Antwis R. E. Haworth R. L. Engelmoer D. J. Ogilvy V. Fidgett A. L. Preziosi R. F. (2014). *Ex situ* diet influences the bacterial community associated with the skin of red-eyed tree frogs (*Agalychnis callidryas*). *PLOS ONE* 9:e85563. 10.1371/journal.pone.0085563 24416427 PMC3887054

[B5] Augustinos A. A. Kyritsis G. A. Papadopoulos N. T. Abd-Alla A. M. M. Caceres C. Bourtzis K. (2015). Exploitation of the medfly gut microbiota for the enhancement of sterile insect technique: use of *Enterobacter* sp. in larval diet-based probiotic applications. *PLOS ONE* 10:e0136459. 10.1371/journal.pone.0136459 26325068 PMC4556606

[B6] Ben Ami E. Yuval B. Jurkevitch E. (2010). Manipulation of the microbiota of mass-reared mediterranean fruit flies *Ceratitis capitata* (Diptera: Tephritidae) improves sterile male sexual performance. *ISME J.* 4 28–37. 10.1038/ismej.2009.82 19617877

[B7] Berasategui A. Shukla S. Salem H. Kaltenpoth M. (2016). Potential applications of insect symbionts in biotechnology. *Appl. Microbiol. Biotechnol.* 100 1567–1577. 10.1007/s00253-015-7186-9 26659224 PMC4737797

[B8] Billiet A. Meeus I. Van Nieuwerburgh F. Deforce D. Wackers F. Smagghe G. (2017). Colony contact contributes to the diversity of gut bacteria in bumblebees (*Bombus terrestris*). *Insect. Sci.* 24 270–277. 10.1111/1744-7917.12284 26503284

[B9] Bokulich N. A. Subramanian S. Faith J. J. Gevers D. Gordon J. I. Knight R. (2013). Quality-filtering vastly improves diversity estimates from Illumina amplicon sequencing. *Nat. Methods.* 10 57–59. 10.1038/nmeth.2276 23202435 PMC3531572

[B10] Caporaso J. G. Kuczynski J. Stombaugh J. Bittinger K. Bushman F. D. Costello E. K. (2010). QIIME allows analysis of high-throughput community sequencing data. *Nat. Methods.* 7 335–336. 10.1038/nmeth.f.303 20383131 PMC3156573

[B11] Cheng D. Guo Z. Riegler M. Xi Z. Liang G. Xu Y. (2017). Gut symbiont enhances insecticide resistance in a significant pest, the oriental fruit fly *Bactrocera dorsalis* (Hendel). *Microbiome* 5:13. 10.1186/s40168-017-0236-z 28143582 PMC5286733

[B12] Dettner K. (2011). “Potential pharmaceuticals from insects and their co-occurring microorganisms,” in *Insect Biotechnology. Biologically-Inspired Systems* Vol. 2 ed. Vilcinskas A. (Dordrecht: Springer).

[B13] Douglas A. E. (2009). The microbial dimension in insect nutritional ecology. *Funct. Ecol.* 23 38–47. 10.1111/j.1365-2435.2008.01442.x

[B14] Douglas A. E. (2015). Multiorganismal insects: diversity and function of resident microorganisms. *Annu. Rev. Entomol.* 60 17–34. 10.1146/annurev-ento-010814-020822 25341109 PMC4465791

[B15] Edgar R. C. (2010). Search and clustering orders of magnitude faster than BLAST. *Bioinformatics* 26 2460–2461. 10.1093/bioinformatics/btq461 20709691

[B16] Egert M. Marhan S. Wagner B. Scheu S. Friedrich M. W. (2004). Molecular profiling of 16S rRNA genes reveals diet-related differences of microbial communities in soil, gut, and casts of *Lumbricus terrestris* L. (Oligochaeta: Lumbricidae). *FEMS Microbiol. Ecol.* 48 187–197. 10.1016/j.femsec.2004.01.007 19712402

[B17] Florez L. V. Biedermann P. H. Engl T. Kaltenpoth M. (2015). Defensive symbioses of animals with prokaryotic and eukaryotic microorganisms. *Nat. Prod. Rep.* 32 904–936. 10.1039/c5np00010f 25891201

[B18] Fredenhagen A. Tamura S. Y. Kenny P. T. M. Komura H. Naya Y. Nakanishi K. (1987). Andrimid, a new peptide antibiotic produced by an intracellular bacterial symbiont isolated from a brown planthopper. *J. Am. Chem. Soc.* 109 4409–4411. 10.1021/ja00248a055

[B19] Goodfellow M. Williams S. T. (1983). Ecology of *actinomycetes*. *Annu. Rev. Microbiol.* 37 189–216. 10.1146/annurev.mi.37.100183.001201 6357051

[B20] Gujjar N. R. Govindan S. Verghese A. Subramaniam S. More R. (2017). Diversity of the cultivable gut bacterial communities associated with the fruit flies *Bactrocera dorsalis* and *Bactrocera cucurbitae* (Diptera: Tephritidae). *Phytoparasitica* 45 453–460. 10.1007/s12600-017-0604-z

[B21] Hamby K. A. Becher P. G. (2016). Current knowledge of interactions between *Drosophila suzukii* and microbes, and their potential utility for pest management. *J. Pest. Sci.* 89 621–630. 10.1007/s10340-016-0768-1

[B22] Hamden H. Guerfali M. M. Fadhl S. Saidi M. Chevrier C. (2013). Fitness improvement of mass-reared sterile males of *Ceratitis capitata* (Vienna 8 strain) (Diptera: Tephritidae) after gut enrichment with probiotics. *J. Econ. Entomol.* 106 641–647. 10.1603/EC12362 23786049

[B23] Huse S. M. Welch D. M. Morrison H. G. Sogin M. L. (2010). Ironing out the wrinkles in the rare biosphere through improved OTU clustering. *Environ. Microbiol.* 12 1889–1898. 10.1111/j.1462-2920.2010.02193.x 20236171 PMC2909393

[B24] Kaltenpoth M. (2009). *Actinobacteria* as mutualists: general healthcare for insects? *Trends Microbiol.* 17 529–535. 10.1016/j.tim.2009.09.006 19853457

[B25] Kaltenpoth M. Engl T. (2014). Defensive microbial symbionts in Hymenoptera. *Funct. Ecol.* 28 315–327. 10.1111/1365-2435.12089

[B26] Kaltenpoth M. Gottler W. Herzner G. Strohm E. (2005). Symbiotic bacteria protect wasp larvae from fungal infestation. *Curr. Biol.* 15 475–479. 10.1016/j.cub.2004.12.084 15753044

[B27] Kellner R. L. (2002). Molecular identification of an endosymbiotic bacterium associated with pederin biosynthesis in *Paederus sabaeus* (Coleoptera: Staphylinidae). *Insect. Biochem. Mol. Biol.* 32 389–395. 10.1016/S0965-1748(01)00115-1 11886773

[B28] Kersters K. Lisdiyanti P. Komagata K. Swings J. (2006). “The family *Acetobacteraceae*: the Genera *Acetobacter, Acidomonas, Asaia, Gluconacetobacter, Gluconobacter*, and *Kozakia*,” in *The Prokaryotes* eds Dworkin M. Falkow S. Rosenberg K. H. Schleifer E. Stackebrandt E. (New York, NY: Springer).

[B29] Khaeso K. Andongma A. A. Akami M. Souliyanonh B. Zhu J. Krutmuang P. (2017). Assessing the effects of gut bacteria manipulation on the development of the oriental fruit fly, *Bactrocera dorsalis* (Diptera; Tephritidae). *Symbiosis* 74 97–105. 10.1007/s13199-017-0493-4

[B30] Knief C. Delmotte N. Chaffron S. Stark M. Innerebner G. Wassmann R. (2012). Metaproteogenomic analysis of microbial communities in the phyllosphere and rhizosphere of rice. *ISME J.* 6 1378–1390. 10.1038/ismej.2011.192 22189496 PMC3379629

[B31] Kueneman J. G. Parfrey L. W. Woodhams D. C. Archer H. M. Knight R. McKenzie V. J. (2014). The amphibian skin-associated microbiome across species, space and life history stages. *Mol. Ecol.* 23 1238–1250. 10.1111/mec.12510 24171949

[B32] Lambert M. Surhone M. T. Tennoe S. F. (2011). *Lactobacillaceae.* Missoula, MT: Betascript Publishing.

[B33] Langille M. G. I. Zaneveld J. Caporaso J. G. McDonald D. Knights D. Reyes J. A. (2013). Predictive functional profiling of microbial communities using 16S rRNA marker gene sequences. *Nat. Biotechnol.* 31 814–821. 10.1038/nbt.2676 23975157 PMC3819121

[B34] Lindow S. E. Brandl M. T. (2003). Microbiology of the phyllosphere. *Appl. Environ. Microbiol.* 69 1875–1883. 10.1128/AEM.69.4.1875-1883.2003 12676659 PMC154815

[B35] Liu Z. X. Huang K. Xiao H. D. Chen Q. H. He J. W. Chen Y. G. (2011). Screening and preliminary identification of the antimicrobial strains associated with *Anthopleura xanthogrammica*. *Chin. J. Antibiot.* 36 416–420.

[B36] Loudon A. H. Woodhams D. C. Parfrey L. W. Archer H. Knight R. McKenzie V. (2014). Microbial community dynamics and effect of environmental microbial reservoirs on red-backed salamanders (*Plethodon cinereus*). *ISME J.* 8 830–840. 10.1038/ismej.2013.200 24335825 PMC3960541

[B37] Maisnierpatin S. Richard J. (1995). Activity and purification of linenscin OC2, an antibacterial substance produced by *Brevibacterium* linens OC2, an orange cheese coryneform bacterium. *Appl. Environ. Microbiol.* 61 1847–1852. 7646021 10.1128/aem.61.5.1847-1852.1995 PMC167446

[B38] Michael E. Shay S. Orit S. Yulia G. Yael H. Yoav S. (2016). Impact of gut microbiota on the fly’s germ line. *Nat. Commun.* 7:11280. 10.1038/ncomms11280 27080728 PMC4835552

[B39] Nakabachi A. Ueoka R. Oshima K. Teta R. Mangoni A. Gurgui M. (2013). Defensive bacteriome symbiont with a drastically reduced genome. *Curr. Biol.* 23 1478–1484. 10.1016/j.cub.2013.06.027 23850282

[B40] Navas-Molina J. A. Peralta-Sanchez J. M. Gonzalez A. McMurdie P. J. Vazquez-Baeza Y. Xu Z. J. (2013). Advancing our understanding of the human microbiome using QIIME. *Methods Enzymol.* 531 371–444. 10.1016/B978-0-12-407863-5.00019-8 24060131 PMC4517945

[B41] Newell P. D. Chaston J. M. Wang Y. Winans N. J. Sannino D. R. Wong A. C. (2014). *In vivo* function and comparative genomic analyses of the *Drosophila* gut microbiota identify candidate symbiosis factors. *Front. Microbiol.* 5:576 10.3389/fmicb.2014.00576 PMC4219406 25408687

[B42] Ohkuma M. Brune A. (2011). “Diversity, structure, and evolution of the termite gut microbial community,” in *Biology of Termites: A Modern Synthesis* eds Bignell D. E. Roisin Y. Lo N. (Dordrecht: Springer) 413–438.

[B43] Oksanen J. Blanchet F. G. Kindt R. Legendre P. Minchin P. R. O’Hara R. B. (2015). *vegan: Community Ecology Package. R Package Version 2.3–1*.

[B44] Rafael J. V. Natalia G. J. Gloria C.-R. Sandra I. U. S. Claudia Ximena M. H. (2016). Structural differences in gut bacteria communities in developmental stages of natural populations of *Lutzomyia evansi* from Colombia’s Caribbean coast. *Parasit. Vectors* 9:496. 10.1186/s13071-016-1766-0 27618991 PMC5020466

[B45] Ramadhar T. R. Beemelmanns C. Currie C. R. Clardy J. (2014). Bacterial symbionts in agricultural systems provide a strategic source for antibiotic discovery. *J. Antibiot.* 67 53–58. 10.1038/ja.2013.77 23921819 PMC7381487

[B46] Rebollar E. A. Hughey M. C. Medina D. Harris R. N. Ibanez R. Belden L. K. (2016). Skin bacterial diversity of Panamanian frogs is associated with host susceptibility and presence of *Batrachochytrium dendrobatidis*. *ISME J.* 10 1682–1695. 10.1038/ismej.2015.234 26744810 PMC4918441

[B47] Rio R. V. Wu Y. N. Filardo G. Aksoy S. (2006). Dynamics of multiple symbiont density regulation during host development: tsetse fly and its microbial flora. *Proc. Biol. Sci.* 273 805–814. 10.1098/rspb.2005.3399 16618673 PMC1560226

[B48] Ryser E. T. Maisnier-Patin S. Gratadoux J. J. Richard J. (1994). Isolation and identification of cheese-smear bacteria inhibitory to *Listeria* spp. *Int. J. Food. Microbiol.* 21 237–246. 10.1016/0168-1605(94)90030-2 8024975

[B49] Schloss P. D. Westcott S. L. Ryabin T. Hall J. R. Hartmann M. Hollister E. B. (2009). Introducing mothur: open-source, platform-independent, community-supported software for describing and comparing microbial communities. *Appl. Environ. Microbiol.* 75 7537–7541. 10.1128/AEM.01541-09 19801464 PMC2786419

[B50] Sharon G. Segal D. Ringo J. M. Hefetz A. Zilber-Rosenberg I. Rosenberg E. (2010). Commensal bacteria play a role in mating preference of *Drosophila melanogaster*. *Proc. Natl. Acad. Sci. U.S.A.* 107 20051–20056. 10.1073/pnas.1009906107 21041648 PMC2993361

[B51] Shelly T. E. Edu J. Pahio E. (2005). Influence of diet and methyl eugenol on the mating success of males of the oriental fruit fly, *Bactrocera dorsalis* (Diptera : Tephritidae). *Fla. Entomol.* 88 307–313. 10.1653/0015-4040(2005)088[0307:IODAME]2.0.CO;2

[B52] Shelly T. E. Kennelly S. (2002). Influence of male diet on male mating success and longevity and female remating in the Mediterranean fruit fly (Diptera : Tephritidae) under laboratory conditions. *Fla. Entomol.* 85 572–579. 10.1653/0015-4040(2002)085[0572:Iomdom]2.0.Co;2

[B53] Teixeira L. Ferreira A. Ashburner M. (2008). The bacterial symbiont *Wolbachia* induces resistance to RNA viral infections in *Drosophila melanogaster*. *PLOS Biol.* 6:e2. 10.1371/journal.pbio.1000002 19222304 PMC2605931

[B54] Turnbaugh P. J. Ley R. E. Hamady M. Fraserliggett C. Knight R. Gordon J. I. (2007). The human microbiome project: exploring the microbial part of ourselves in a changing world. *Nature* 449 804–810. 10.1038/nature06244 17943116 PMC3709439

[B55] Vänninen I. Tynijuslin J. Hokkanen H. (2000). Persistence of augmented *Metarhizium anisopliae* and *Beauveria bassiana* in Finnish agricultural soils. *Biocontrol* 45 201–222. 10.1023/A:1009998919531

[B56] Wang H. Jin L. Zhang H. (2011). Comparison of the diversity of the bacterial communities in the intestinal tract of adult *Bactrocera dorsalis* from three different populations. *J. Appl. Microbiol.* 110 1390–1401. 10.1111/j.1365-2672.2011.05001.x 21395953

[B57] Wang H. X. Jin L. Peng T. Zhang H. Y. Chen Q. L. Hua Y. J. (2014). Identification of cultivable bacteria in the intestinal tract of *Bactrocera dorsalis* from three different populations and determination of their attractive potential. *Pest. Manag. Sci.* 70 80–87. 10.1002/ps.3528 23696217

[B58] Ye L. Amberg J. Chapman D. Gaikowski M. Liu W. T. (2014). Fish gut microbiota analysis differentiates physiology and behavior of invasive Asian carp and indigenous American fish. *ISME J.* 8 541–551. 10.1038/ismej.2013.181 24132079 PMC3930320

[B59] Yong H. S. Song S. L. Chua K. O. Lim P. E. (2017a). High diversity of bacterial communities in developmental stages of *Bactrocera carambolae* (Insecta: Tephritidae) revealed by Illumina MiSeq sequencing of 16S rRNA gene. *Curr. Microbiol.* 74 1076–1082. 10.1007/s00284-017-1287-x 28642971

[B60] Yong H. S. Song S. L. Chua K. O. Lim P. E. (2017b). Microbiota associated with *Bactrocera carambolae* and B. dorsalis (Insecta: Tephritidae) revealed by next-generation sequencing of 16S rRNA gene. *Meta Gene* 11 189–196. 10.1016/j.mgene.2016.10.009

[B61] Zhang J. Xia Z. He J. Sun H. Zhang L. (2013). Evaluation outcome of *actinobacteria* diversity in saline environment influenced by different DNA extraction methods. *Wei Sheng Wu Xue Bao* 53 746–757. 24195382

[B62] Zhao Y. Wang W. Q. Zhu F. Wang X. Y. Wang X. P. Lei C. L. (2017). The gut microbiota in larvae of the housefly *Musca domestica* and their horizontal transfer through feeding. *AMB Express* 7:147. 10.1186/S13568-017-0445-7 28697583 PMC5503848

