# Supplementary figures and images for: The Divergence in Bacterial Components Associated with Bactrocera dorsalis across Developmental Stages
Source: Front Microbiol. 2018 Feb 1;9:114. doi: 10.3389/fmicb.2018.00114 (PMC5799270; doi:10.3389/fmicb.2018.00114)

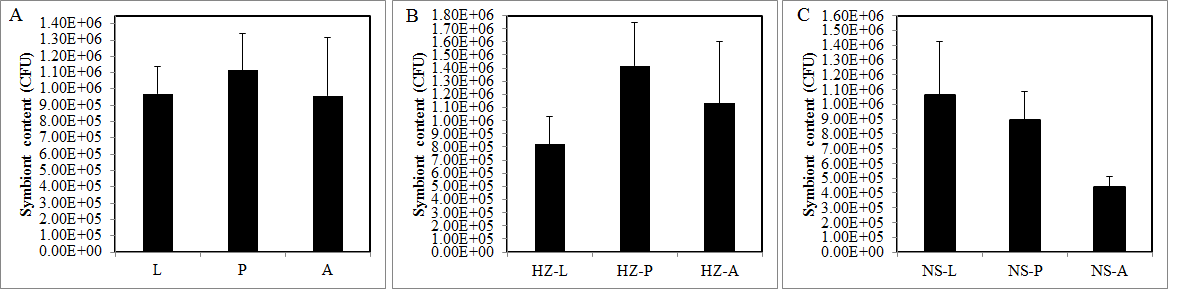

Supplement: FIGURE S1 — Absolute content of symbionts in flies. [file Image_1.TIF]

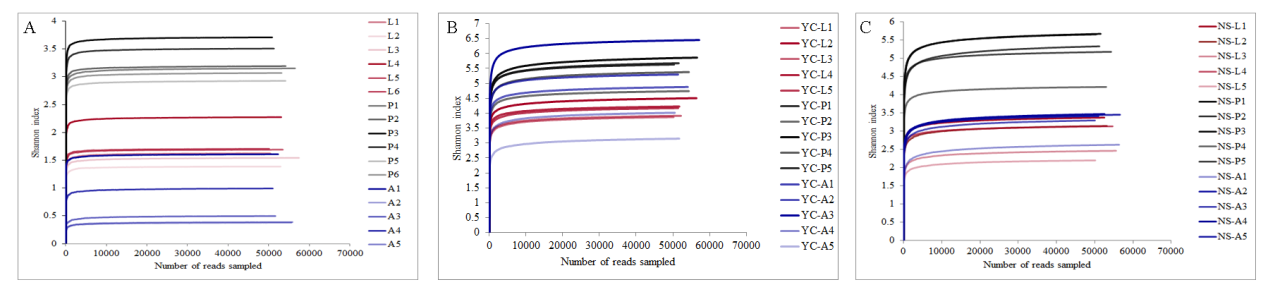

Supplement: FIGURE S2 — Shannon rarefaction curves for all samples. [file Image_2.TIF]
