# Supplementary material for: The Divergence in Bacterial Components Associated with Bactrocera dorsalis across Developmental Stages
Source: Front Microbiol. 2018 Feb 1;9:114. doi: 10.3389/fmicb.2018.00114 (PMC5799270; doi:10.3389/fmicb.2018.00114)
Supplement: TABLE S1 — Summary of 16S rDNA read counts for all samples of B. dorsalis. [file Table_1.DOCX]

Table S1 Summary of 16S rDNA read counts for all samples of *B.dorsalis*

| SampleID | Reads number | Total length | Max length | Min length | N50 | N90 |
| --- | --- | --- | --- | --- | --- | --- |
| L1 | 50376 | 22647141 | 478 | 306 | 441 | 441 |
| L2 | 52918 | 23673947 | 479 | 307 | 441 | 441 |
| L3 | 57433 | 25820342 | 479 | 343 | 441 | 441 |
| L4 | 53064 | 23976912 | 479 | 353 | 442 | 441 |
| L5 | 53454 | 24068807 | 479 | 353 | 441 | 441 |
| L6 | 50082 | 22535586 | 479 | 328 | 441 | 441 |
| P1 | 56471 | 25508372 | 470 | 409 | 446 | 446 |
| P2 | 54149 | 24595275 | 469 | 419 | 447 | 446 |
| P3 | 50833 | 22989212 | 470 | 423 | 447 | 442 |
| P4 | 51252 | 23222090 | 477 | 329 | 446 | 441 |
| P5 | 54121 | 24720779 | 470 | 425 | 466 | 446 |
| P6 | 53152 | 24208381 | 479 | 412 | 466 | 441 |
| A1 | 52300 | 24341278 | 477 | 438 | 466 | 466 |
| A2 | 55596 | 25889910 | 468 | 385 | 466 | 466 |
| A3 | 51626 | 24032806 | 468 | 351 | 466 | 466 |
| A4 | 50995 | 23719212 | 478 | 351 | 466 | 466 |
| A5 | 55842 | 26011434 | 469 | 429 | 466 | 466 |
| YC-L1 | 50176 | 22953285 | 477 | 324 | 466 | 441 |
| YC-L2 | 56428 | 25585673 | 477 | 429 | 461 | 441 |
| YC-L3 | 52191 | 23604187 | 477 | 409 | 441 | 441 |
| YC-L4 | 51751 | 23240396 | 477 | 374 | 441 | 441 |
| YC-L5 | 51433 | 23336233 | 477 | 398 | 463 | 441 |
| YC-P1 | 50301 | 22737927 | 477 | 422 | 447 | 441 |
| YC-P2 | 54344 | 24319049 | 477 | 330 | 441 | 441 |
| YC-P3 | 56593 | 25707336 | 477 | 436 | 460 | 441 |
| YC-P4 | 54358 | 24740163 | 477 | 421 | 460 | 441 |
| YC-P5 | 51582 | 23496658 | 477 | 342 | 461 | 441 |
| YC-A1 | 51409 | 23307642 | 477 | 346 | 448 | 441 |
| YC-A2 | 54058 | 24478924 | 477 | 354 | 448 | 441 |
| YC-A3 | 57172 | 25872909 | 477 | 429 | 447 | 441 |
| YC-A4 | 50522 | 23397342 | 477 | 436 | 466 | 459 |
| YC-A5 | 51737 | 24012535 | 477 | 436 | 466 | 465 |
| NS-L1 | 52498 | 23415367 | 477 | 331 | 441 | 441 |
| NS-L2 | 53146 | 23678480 | 477 | 361 | 441 | 441 |
| NS-L3 | 55551 | 24652451 | 477 | 325 | 441 | 441 |
| NS-L4 | 54666 | 24278009 | 477 | 371 | 441 | 441 |
| NS-L5 | 50127 | 22217844 | 477 | 435 | 441 | 441 |
| NS-P1 | 50349 | 22812904 | 477 | 346 | 447 | 441 |
| NS-P2 | 51203 | 23511273 | 477 | 373 | 466 | 443 |
| NS-P3 | 51543 | 23343101 | 477 | 360 | 447 | 441 |
| NS-P4 | 52977 | 24390924 | 477 | 430 | 466 | 441 |
| NS-P5 | 54270 | 24540839 | 477 | 383 | 447 | 441 |
| NS-A1 | 56363 | 26231867 | 477 | 439 | 466 | 465 |
| NS-A2 | 56599 | 26320289 | 477 | 411 | 466 | 465 |
| NS-A3 | 50037 | 23306213 | 477 | 390 | 466 | 466 |
| NS-A4 | 52552 | 24473037 | 477 | 439 | 466 | 466 |
| NS-A5 | 50966 | 23736111 | 477 | 439 | 466 | 466 |
