# Supplementary material for: The Divergence in Bacterial Components Associated with Bactrocera dorsalis across Developmental Stages
Source: Front Microbiol. 2018 Feb 1;9:114. doi: 10.3389/fmicb.2018.00114 (PMC5799270; doi:10.3389/fmicb.2018.00114)
Supplement: TABLE S2 — Comparison of OTU diversity of 16S rRNA gene amplicons. [file Table_2.DOCX]

Table S2 Comparison of OTU diversity of 16S rRNA gene amplicons

| Sample | Sample size | ACE (SD) | Shannon (SD) | Npshannon (SD) | Simpson (SD) |
| --- | --- | --- | --- | --- | --- |
| Lab population | |  |  |  |  |
| L | 6 | 3427.2(428.6) | 1.7(0.30) | 1.75(0.31) | 0.37(0.08) |
| P | 6 | 5239.8(1236.9)** | 3.26(0.29) ** | 3.31(0.29) ** | 0.09(0.02) ** |
| A | 5 | 3336.6(479.9) | 0.77(0.53) | 0.83(0.53) | 0.76(0.24) |
| Huizhou population | |  |  |  |  |
| L | 5 | 40823.9(11393.1) | 4.14(0.26) | 4.43(0.25) | 0.06(0.02) |
| P | 5 | 65990.7(11644.02) ** | 5.46(0.44) ** | 5.72(0.44) ** | 0.03(0.01) ** |
| A | 5 | 47366.5(19723.79) | 4.76(1.26) | 5.04(1.17) | 0.08(0.10) |
| Nanshan population | |  |  |  |  |
| L | 5 | 30203.7(6302.6) | 2.86(0.5) | 3.15(0.52) | 0.31(0.11) |
| P | 5 | 50331.7(19192.09) ** | 5.21(0.6) ** | 5.46(0.63) ** | 0.03(0.01) ** |
| A | 5 | 27805.3(3766.62) | 3.25(0.36) | 3.61(0.33) | 0.21(0.13) |

Note: ** indicates that the values were significantly different from the others within each population (*P* < 0.01)
